# The challenges of introducing routine G6PD testing into radical cure: a workshop report

**DOI:** 10.1186/s12936-015-0896-8

**Published:** 2015-09-29

**Authors:** Benedikt Ley, Nick Luter, Fe Esperanza Espino, Angela Devine, Michael Kalnoky, Yoel Lubell, Kamala Thriemer, J. Kevin Baird, Eugenie Poirot, Nolwenn Conan, Chong Chee Kheong, Lek Dysoley, Wasif Ali Khan, April G. Dion-Berboso, Germana Bancone, Jimee Hwang, Ritu Kumar, Ric N. Price, Lorenz von Seidlein, Gonzalo J. Domingo

**Affiliations:** Global and Tropical Health Division, Menzies School of Health Research, Charles Darwin University, PO Box 41096, Casuarina, Darwin, NT 0811 Australia; PATH, Diagnostics Program, Seattle, WA USA; Research Institute of Tropical Medicine, Manila, Philippines; Mahidol-Oxford Tropical Medicine Research Unit, Mahidol University, Bangkok, Thailand; Eijkman-Oxford Clinical Research Unit, Jakarta, Indonesia; Nuffield Department of Medicine, Centre for Tropical Medicine and Global Health, University of Oxford, Oxford, UK; The Malaria Elimination Initiative, Global Health Group, University of California, San Francisco, CA USA; Malaria Consortium, Bangkok, Thailand; Disease Control Division, Ministry of Health Malaysia, Kuala Lumpur, Malaysia; National Center for Parasitology Entomology and Malaria Control, Phnom Penh, Cambodia; School of Public Health, National Institution of Public Health, Phnom Penh, Cambodia; International Center for Diarrheal Disease Research, Bangladesh (icddr, b), Dhaka, Bangladesh; Newborn Screening Center, Institute of Human Genetics, National Institutes of Health, University of the Philippines, Manila, Philippines; Shoklo Malaria Research Unit, Mae Sot, Tak Province Thailand; Malaria Branch, Division of Parasitic Diseases and Malaria, Centers for Disease Control and Prevention, Atlanta, Georgia USA

**Keywords:** G6PD, Point of care test, *Plasmodium vivax*, Primaquine, Malaria

## Abstract

The only currently available drug that effectively removes malaria hypnozoites from the human host is primaquine. The use of 8-aminoquinolines is hampered by haemolytic side effects in glucose-6-phosphate dehydrogenase (G6PD) deficient individuals. Recently a number of qualitative and a quantitative rapid diagnostic test (RDT) format have been developed that provide an alternative to the current standard G6PD activity assays. The WHO has recently recommended routine testing of G6PD status prior to primaquine radical cure whenever possible. A workshop was held in the Philippines in early 2015 to discuss key challenges and knowledge gaps that hinder the introduction of routine G6PD testing. Two point-of-care (PoC) test formats for the measurement of G6PD activity are currently available: qualitative tests comparable to malaria RDT as well as biosensors that provide a quantitative reading. Qualitative G6PD PoC tests provide a binomial test result, are easy to use and some products are comparable in price to the widely used fluorescent spot test. Qualitative test results can accurately classify hemizygous males, heterozygous females, but may misclassify females with intermediate G6PD activity. Biosensors provide a more complex quantitative readout and are better suited to identify heterozygous females. While associated with higher costs per sample tested biosensors have the potential for broader use in other scenarios where knowledge of G6PD activity is relevant as well. The introduction of routine G6PD testing is associated with additional costs on top of routine treatment that will vary by setting and will need to be assessed prior to test introduction. Reliable G6PD PoC tests have the potential to play an essential role in future malaria elimination programmes, however require an improved understanding on how to best integrate routine G6PD testing into different health settings.

**Electronic supplementary material:**

The online version of this article (doi:10.1186/s12936-015-0896-8) contains supplementary material, which is available to authorized users.

## Introduction

*Plasmodium vivax* has long been considered a benign form of malaria, but this paradigm is changing. Vivax malaria can have a profound impact on health, particularly in women and children from poorly resourced communities [1–3]. Successful control and ultimate elimination of *P. vivax* will require a radical cure, that combines a schizontocide to eliminate blood stages of the parasite and a hypnozoiticide to kill the liver stages. Primaquine, an 8-aminoquinoline (8-AQ), is the only widely available hypnozoiticide [4], although a new, slowly eliminated 8-aminoquinoline, tafenoquine is currently in Phase III clinical trials [5]. Although 8-aminoquinolones are well tolerated by most individuals [6], this class of drugs can trigger haemolytic reactions in glucose-6-phosphate dehydrogenase (G6PD) deficient recipients [7]. The risk of drug induced haemolysis in relatively small G6PD deficient subpopulations needs to be balanced with the risk of anaemia and other detrimental effects due to recurrent episodes of malaria in all vivax patients. Reliable, easy to perform point-of-care (PoC) tests provide a potential solution to this dilemma. If G6PD status can be assessed before commencing radical cure treatment, the threat of severe side effects from primaquine can be minimized in the at-risk population.

For several decades the research agenda for radical cure has been neglected and the demand for reliable, easy-to-use G6PD tests has not been a priority. In the last decade malaria control and elimination programmes have gained considerable momentum, particularly in reducing the burden of *P. falciparum.* Increasingly the need for an improved management of *P. vivax* is recognized as a programmatic priority. Based on the experience in G6PD deficient African American soldiers, radical cure with primaquine was long considered safe; however, the potential risks in more severe variants of G6PD deficiency can be considerable. In the most recent WHO treatment guidelines G6PD testing to guide radical cure is recommended whenever possible [8]. When testing is not possible the risks and benefits of primaquine treatment must be weighed prior to drug administration. The administration of a single low dose of 0.25 mg/kg bw primaquine to kill *P. falciparum* gametocytes is considered unlikely to cause serious toxicity even in people with G6PD deficiency [8] and G6PD testing is not recommended prior to the administration of a single low dose.

Since 2012 a series of workshops have been held by the Asia Pacific Malaria Elimination Network (APMEN) to discuss G6PD testing in the context of vivax malaria treatment in the Asia Pacific. The first workshop of this series was held in Incheon, South Korea in May 2012, where the research agenda for G6PD deficiency and the radical cure of *P. vivax* was reviewed, an update of available tests presented, and a target product profile for a point-of-care test for G6PD deficiency proposed [7]. The focus of the second workshop in October 2012 in Bangkok, Thailand was on use—case scenarios, updating the target product profile for point-of-care tests, and discussing criteria for their evaluation [9].

This article reports on the third workshop held at the Research Institute of Tropical Medicine (RITM), the Philippines, in February 2015, in a joint effort by the Asia Pacific Malaria Elimination Network (APMEN), RITM and PATH. Main objectives of the workshop were on the challenges and evidence gaps facing successful implementation of G6PD screening in the context of currently available and anticipated point-of-care tests for G6PD deficiency. The workshop included participants from Australia, Bangladesh, Cambodia, Indonesia, Malaysia, the Philippines, Thailand, UK, US and Vietnam, representing research institutions and country malaria control programmes. The discussion was timely in the context of the most recent recommendations emerging from the 2014 WHO expert review group on G6PD deficiency [10], the resulting guidance from the March 2015 WHO Malaria Policy Advisory Committee and the recently published malaria treatment guidelines by the WHO [8].

## Background

Glucose-6-phosphate dehydrogenase (G6PD) is an essential enzyme in the pentose phosphate pathway which is the sole source of energy for red blood cells (RBCs) and the only mechanism to maintain the cells redox potential [11]. In contrast to other human cells, RBCs do not have a nucleus and are hence reliant on the enzyme molecules provided during erythropoiesis. As a result the life expectancy of healthy RBCs under normal circumstances is comparably short at around 120 days [12].

Intracellular enzyme activity in RBCs is a function of the initial abundance of the enzyme, enzyme half-life and the RBC half-life. G6PD deficiency (G6PDd) is the result of a structural defect of the G6PD enzyme and is one of the most common enzymopathies worldwide [13, 14]. G6PD deficiencies vary from slightly reduced G6PD activities to extremely low G6PD activity even in young RBCs, while the absence of enzyme activity is not compatible with human life [15]. In 1989, the WHO G6PD working group proposed to categorize G6PDd into classes I–V [16] based on measured activity relative to normal G6PD activity (in percent). Defining an absolute quantitative 100 % G6PD activity is challenging due to its dependence on the population under consideration, the assay conditions and assay platform. Current definitions are population specific and based on the median G6PD activity of all males excluding those that are hemizygous G6PD deficient (Table 1) [9].

**Table 1:** Calculating G6PD activity

The WHO has defined a total of five classes (I–V) of G6PD activity [16]:
• Severe deficiency (<10 % activity, chronic, non-spherocytic, haemolytic anaemia)
• Severe deficiency (<10 % activity, intermittent haemolysis)
• Mild deficiency (10–60 % activity, haemolysis with stressors only)
• Normal enzyme variant (60–150 % activity, no clinical sequelae)
• Increased enzyme activity (>150 % activity, no clinical sequelae)
100 % G6PD activity is based on the adjusted quantitative (iU/gHb or U/10^12^ RBC) median of all male samples from a defined sample set [9]. In a first step the median G6PD activity of samples from all male participants is calculated. Second, all samples with ≤10 % G6PD activity of the median are excluded. Third, the median is re-calculated based on the remaining samples, the adjusted male median. The adjusted male median is defined as 100 % G6PD activity and all samples are grouped accordingly

The underlying G6PD gene is located on the X-chromosome (Xq28), spans a total length of 18.5 kb and includes 13 exons and 12 introns [17]. More than 185 G6PD mutations have been described to date [18] giving rise to hemizygous men as well as homozygous and heterozygous women, the latter possessing two distinct populations of RBCs. In heterozygous females G6PD normal and G6PD deficient RBCs co-exist in varying proportions determined by random X-chromosome inactivation (lyonization) [7].

Higher G6PD prevalence rates appear more commonly where higher rates of malaria transmission occur. Such trends may be directly related to protection against severe malaria conferred by some variants of G6PDd [19–22]. G6PDd affects approximately 400 million people worldwide [23]. Manifestations can include neonatal jaundice, favism and haemolytic anemia but in the vast majority of G6PD deficient cases quality of life is not affected and G6PD deficient people may not even be aware of their condition. However in the presence of oxidizing agents the reduced activity of the G6PD enzyme results in a dis-balanced redox equilibrium of the RBC and ultimate destruction of the cell, i.e. haemolysis [21]. Numerous compounds can induce haemolysis in G6PDd RBCs including 8-aminoquinoline based anti-malarial drugs [24].

### Quantitative versus qualitative test formats

Diagnostic assays can be grouped into genotypic assays, sequencing methods as well as phenotypic test assays [7]. Genotypic assays and sequencing methods can provide a precise option for diagnosing G6PD mutations but require long and complicated test procedures, a well-equipped laboratory and highly trained staff [25, 26]. Phenotypic tests can be grouped into qualitative, quantitative and cytochemical test assays. Phenotypic tests are based on the direct or indirect detection of NADPH + H^+^, formed as a result of G6PD activity [11].

Qualitative test formats indicate activity above a test’s inherent activity threshold level. While qualitative test formats are easier to perform and interpret compared to quantitative test methods and cytochemical tests, the reduction of a quantitative phenomenon (G6PD activity) to a binominal outcome is problematic. For technical reasons, current qualitative tests can only accurately diagnose G6PDd in people with G6PD activity below 30–40 % normal activity [27–29]. While this accurately identifies all hemizygous G6PDd males and homozygous G6PDd females, females with heterozygous G6PD alleles are not accurately discriminated. G6PD normal and G6PDd red blood cell (RBC) populations can co-exist within a heterozygous female [7]. In these women, G6PD normal RBCs may mask G6PD deficient cell populations and can result in G6PD normal test results [27, 30–32]. Currently, the relationship between G6PD activity and degree of drug induced haemolysis is poorly understood and varies depending on the haemolytic potential of the applied drug [21].

A quantitative test assay will accommodate different threshold activities and within limits is able to identify heterozygous females as individuals with intermediate G6PD activity (as a result of G6PD normal and G6PD deficient cell populations). Most quantitative test assays to date require a good laboratory infrastructure and well-trained staff. Handheld devices that do not rely on laboratory infrastructure and can provide results within several minutes are currently being introduced but require further evaluation before treatment decisions can be based on these devices.

Only cytochemical assays can effectively distinguish between G6PD normal and G6PD deficient RBCs on a cellular level and can effectively identify heterozygous women with a high percentage of G6PDd red blood cells that are accordingly at risk for severe haemolysis [33]. There are flow cytometry-based assays that allow the measurement of G6PD activity in labelled RBCs [34]. The main drawbacks of this format are the complexity of the respective test assays and interpretation, the need for costly machinery and the long turn-around time that make these assays unsuitable for PoC testing.

### Product options for point-of-care G6PD testing

For many years the Fluorescent Spot Test (FST) was the only available PoC for G6PD testing. The inherent limitations of the FST include the requirement of basic lab facilities (refrigeration, a water bath, and UV light), the qualitative nature of the test and the need for significant upfront training of the test users [35]. In recent years novel technologies are changing the landscape [29, 36, 37]. Included in these new technologies are three PoC devices (Table 2).

**Table 2:** Summary specifications for a subset of commercially available G6PD Tests

Test name	Resource requirements (equipment, infrastructure, etc.).	Kit storage temp. and complexity	Number of steps for sample prep	Total time to result	Through-put capacity	Working temp. ranges	Specimen preservation indications	Price of product
Trinity G-6-PDH (kinetic spectrophotometry)	Electricity, spectrophotometer with temp. control, pipettes, deionized water, timer, water bath (optional)	2°–8 °C and high	8	15–25 min	Low	20°–39 °C	1 week at 2°–8 °C	$10.00*
Trinity fluorescent spot test (FST)	UV light with dark room or viewing box, pipettes, timer	2°–8 °C and moderate	5	25–30 min	Medium	37 °C	1 week at 2°–8 °C	~$3.00
CareStart G6PD screening Test	Pipettes, timer	Not provided and low	3	5–10 min	Medium–high	Not specified	3 days at 2°–8 °C, or frozen	$1.50
BinaxNOW G6PD test	Pipettes, timer	15°–30 °C and low	5	5–7 min	Medium–high	18°–25 °C	Not specified	$20.00
CareStart G6PD biosensor test	Only needle for finger stick	Not provided and low	1	4 min	Medium	Not specified but likely broad	Not specified	$500 for Biosensor, $2.50 per test strip

*Sources:* Price information for Trinity G-6-PDH and CareStart G6PD RDT based on von Fricken et al. [53]. Carestart G6PD RDT and Carestart Biosensor confirmed via correspondence with Accessbio. BinaxNow price is from correspondence with Alere©. Trinity FST is an estimated price per use based on recent PATH purchases. End prices to user will vary widely based on local distributor pricing and in the case of the assay kits (FST and spectrophotometry) individual laboratory sample through put and workflow

* Price varies according to number of samples run/control and number of runs performed/sample

Two companies, Accessbio (New Jersey, USA) and Alere (Maine, USA), have developed different types of PoC diagnostics for G6PD deficiency that focus on rapid diagnosis, with limited need for technical training and infrastructure [28, 35]. Both companies have created qualitative RDTs. In addition to the RDT, CareStart has also produced a quantitative biosensor reader, which is currently under evaluation in several field sites.

The format of the qualitative, lateral flow RDT is similar to many malaria RDTs on the market. In contrast to the detection of Plasmodium antigens the G6PD RDT is based on the reduction of colourless nitro blue tetrazolium dye to dark coloured formazan [38]. The appearance of a purple/blue coloration indicates a G6PD normal result. While the BinaxNOW G6PD test (Alere, USA), available since 2008, has good performance in controlled laboratory settings [27, 37] performance of this test in less controlled settings was less satisfactory [39]. In 2013, Accessbio (USA) released the CareStart G6PD test onto the market. Recent studies have indicated that the CareStart G6PD test is non-inferior in the diagnosis of G6PD deficiency to the FST [28]. Furthermore, field studies in Cambodia have indicated that the CareStart G6PD RDT is capable of reliably detecting G6PD deficient individuals with enzyme activity levels <30 % of normal activity [29]. However, the current Carestart G6PD RDT does not include a control-line, a short-coming that affects result validity.

Biosensors, the second type of PoC device developed, are handheld devices that in conjunction with a disposable strip provide a quantitative result. These tests directly measure G6PD activity from collected blood based on electro-chemical properties of the sample. In 2015, AccessBio (USA) launched the CareStart Biosensor, which at present, is the only product of this type on the market. The test is similar to a glucometer common in many developed country markets. The biosensor has a quantitative readout of G6PD activity and provides a number of advantages over a qualitative RDT, including the possibility to design malaria treatment schemes based on the test readout, the ability to use readouts for drugs outside of 8-aminoquinolines and improved identification of heterozygous females with intermediate G6PD activity. The device has not been validated to date and is limited by the absence of an integrated haemoglobin Hb reader which is required for the estimation of IU/g Hb. These are exciting and over-due new offerings in the G6PD testing product pipeline, but compared to malaria or HIV RDTs, the pipeline remains very thin.

### Favorable environments for different test options

When G6PD status is unknown and G6PD testing is not available, the 2015 WHO treatment guidelines suggest to base the decision to prescribe primaquine on an assessment of the risks and benefits of the treatment [8]. The ethical implications of primaquine therapy against malaria transmission and G6PD testing have been discussed recently and are beyond the scope of this report [40]. The risks of treatment are reduced under several conditions such as a low prevalence of G6PDd, the absence of G6PD variants associated with severe haemolysis [41], and the absence of sub-populations at an increased risk of drug induced haemolysis. The benefits of radical cure are also likely to vary, depending on factors such as the patient profile, as well as the vivax strain and its associated probability and frequency of relapse. Finally, close follow-up of all patients receiving treatment must be feasible and facilities to respond to severe haemolytic reactions must be available. Where the clinical benefits of radical cure are assumed to outweigh the risk of haemolysis, the cost implications of treating rare haemolytic events should also be balanced against the cost savings from relapses avoided.

In reality, even where the risk/benefit ratio and economic considerations would suggest that radical cure is advised without G6PD testing, clinicians often refrain from prescribing primaquine. There is a strong argument therefore to advocate G6PD testing more universally.

**Fig. 1:**
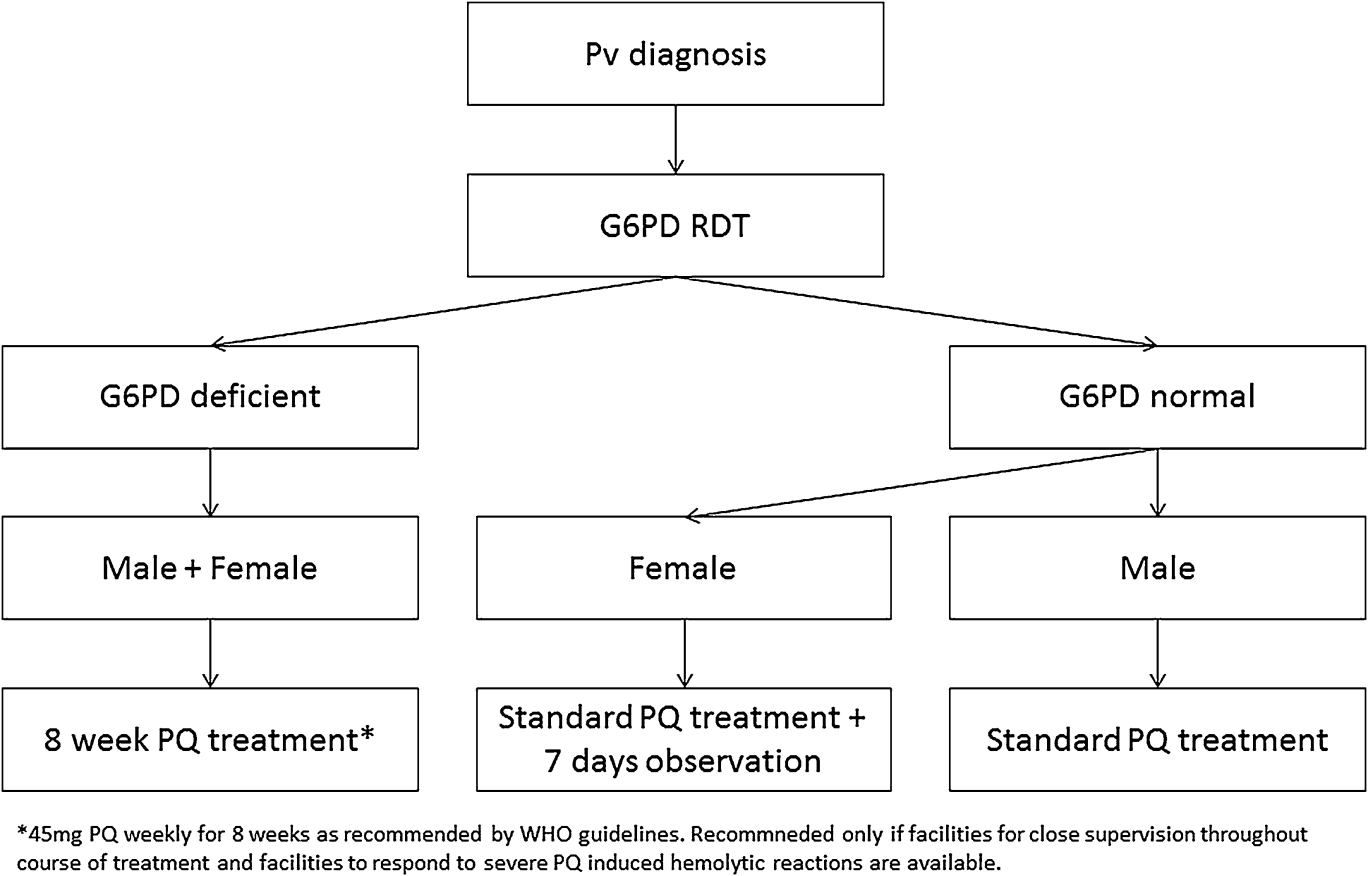
G6PD qualitative RDT only

**Fig. 2:**
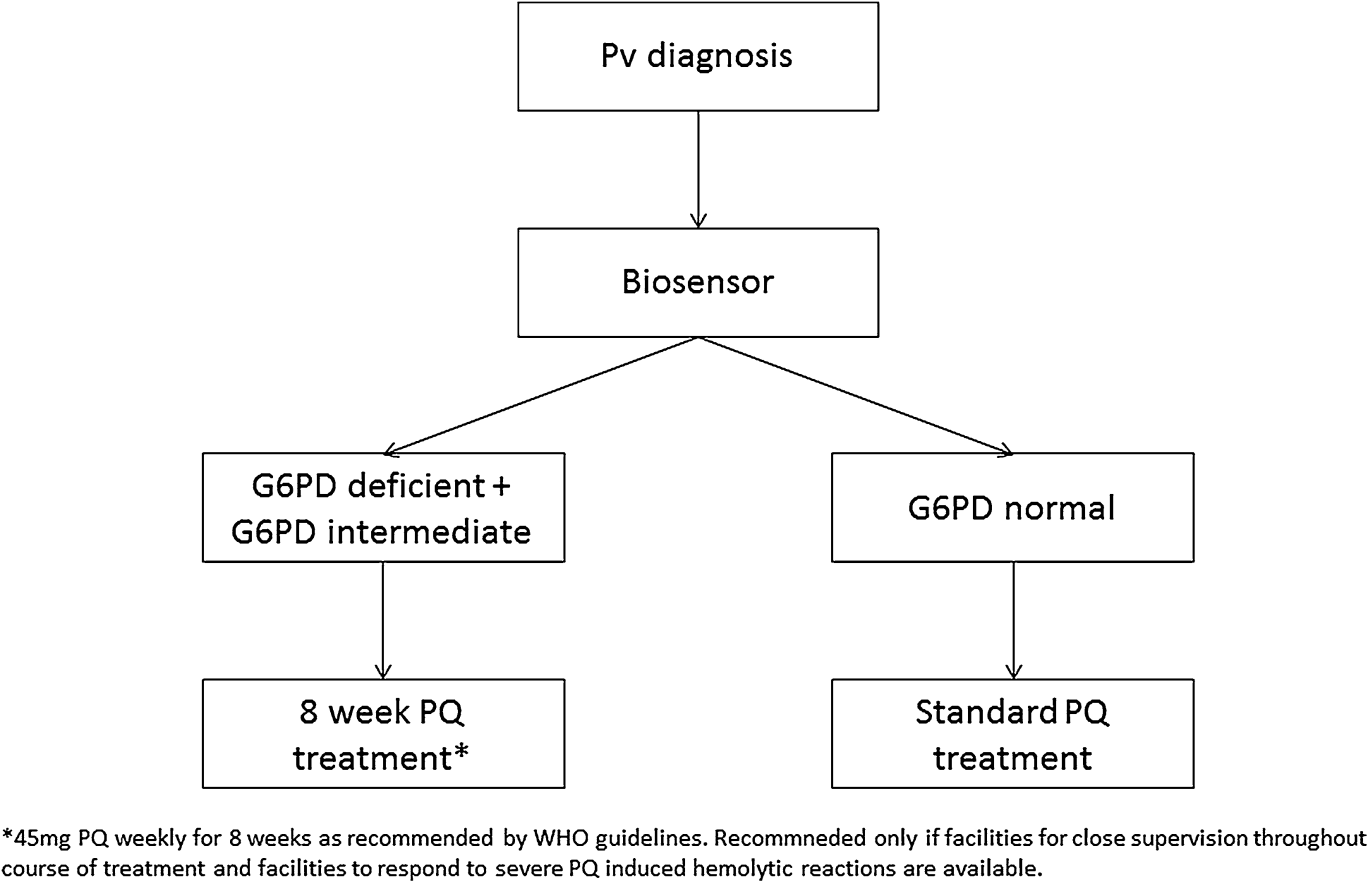
G6PD quantitative biosensor only

**Fig. 3:**
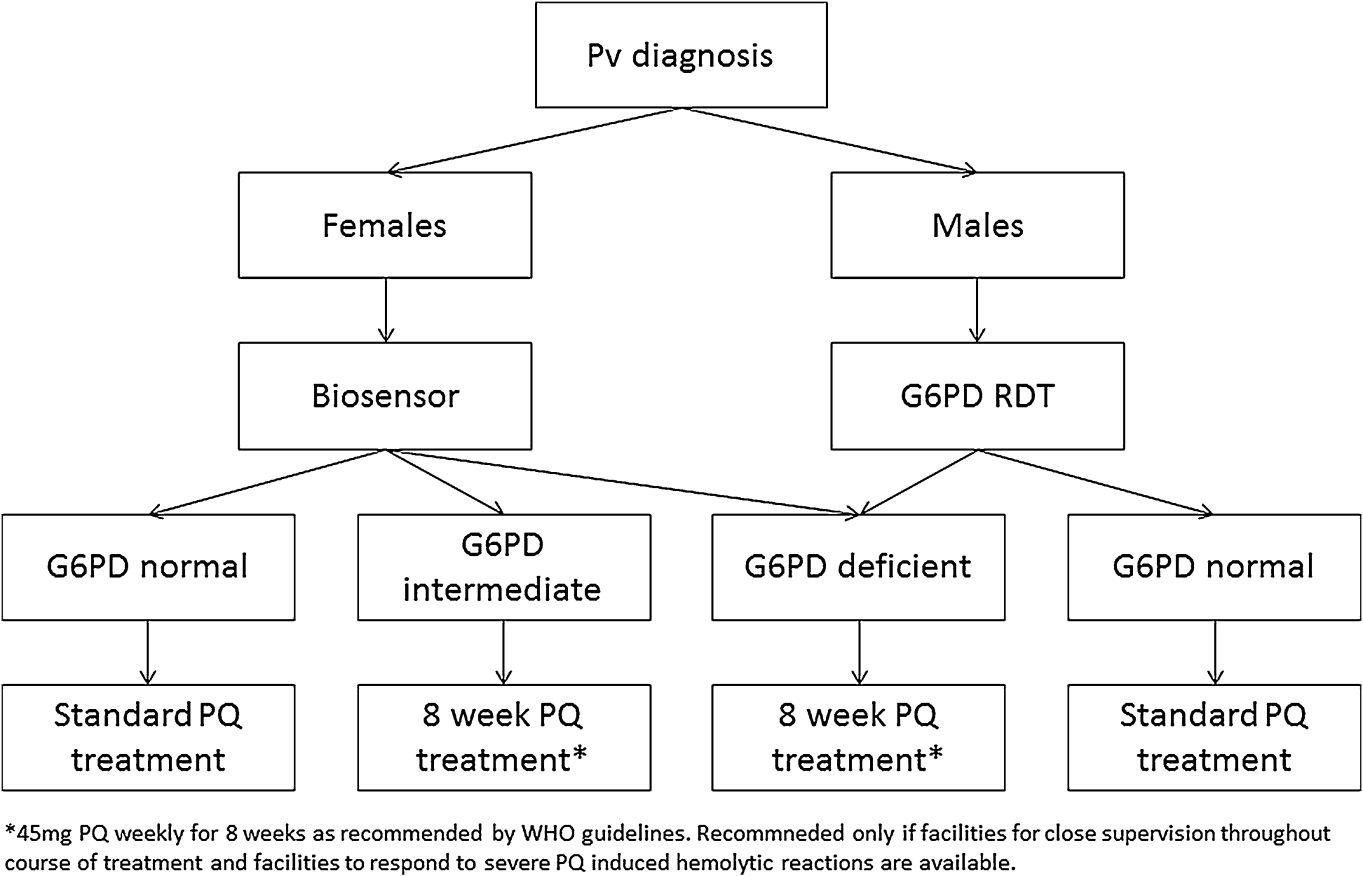
G6PD qualitative RDT and quantitative biosensor

Routine testing using both the RDT and biosensor in point-of-care situations prior to vivax treatment has advantages but also risks. Based on strengths and weaknesses, three test and treat algorithms can be considered (Figs. 1, 2, 3).

#### Scenario 1

G6PD RDT (Fig. 1): The qualitative RDT provides a binary result that can be used at almost any level of the health system and requires little training. The RDT is well suited to discriminate homozygous and hemizygous G6PD deficient and G6PD normal males, but cannot identify heterozygous females, putting these women at increased risk for a potentially false normal G6PD result. All women testing G6PD normal by the RDT treated according to WHO treatment guidelines for radical cure in G6PD normal individuals [8] should be monitored for haemolysis during the first 7 days of treatment [42]; alternatively they can be treated with the 8 week 45 mg primaquine/week regimen as recommended for G6PD deficient individuals [8, 10]. RDT formats with a comparable performance as the FST [28] have the potential to replace the latter in settings where the FST is currently in use due to superior operational characteristics, such as a faster turn-around time and no need for additional instrumentation or refrigeration.

#### Scenario 2

G6PD Biosensor (Fig. 2): The Biosensor can rapidly provide a quantitative result on G6PD activity, and potentially allows identifying heterozygous women. All female patients with a Biosensor based G6PD normal results can undergo radical cure as recommended by the WHO [8] without further precautionary measures. Any patient identified as G6PD intermediate or deficient will require a prolonged course of treatment [8]. While the broad use of the Biosensor may appear ideal considering the increased treatment safety for female patients, it may come at a greater cost and the need additional technical training. In order for the Biosensor to be effective, sound knowledge of G6PD threshold activities for different drugs and G6PD variants, as well as a universally applicable quantitative definition of G6PD deficiency may be needed; however, a hand held device can be programmed to provide a simple interpretation of the result and whether to provide treatment.

#### Scenario 3

Combined use of RDT and Biosensor (Fig. 3): The c ombined product scenario creates the potential for a system in which all male patients are tested with the RDT and the result is directly translated into a radical cure treatment scheme according to WHO guidelines [8]. All female patients are tested with the Biosensor to identify heterozygous females and treated with a prolonged primaquine regimen [8].

Irrespective of the applied test format to determine G6PD status, prolonged primaquine courses are usually reserved for patients with mild to moderate G6PD deficiency and only if appropriate monitoring and facilities are available to respond to severe haemolytic reactions [43].

### Economics of testing for G6PD deficiency

The burden of *P. vivax* is difficult to determine for a variety of reasons. Firstly, cases often happen in areas where there are poor health care systems. Indirect costs of disease include lost productivity and transport to the health care facilities. Each relapse results in at least 3 days off work [1]. Time off work can contribute to poverty and malnutrition. In addition, recurrent disease makes people more vulnerable to anemia and other diseases. It is also challenging to differentiate between primary episodes and relapses; in some areas it has been found that the majority of cases are relapses while in other areas primary infections are the lion’s share of the total burden [44, 45]. This confounds the understanding of the burden of disease as well as the potential impact of interventions to mitigate its effects.

G6PD testing can enable the wider use of primaquine to prevent relapses in areas where it is not used for fear of haemolysis. In areas that give out primaquine without knowledge of G6PD status, G6PD testing can enable the safer use of primaquine. Testing for G6PD deficiency and the selection of a specific test format prior to therapeutic decisions will have cost implications (Additional file 1). In addition to the aforementioned indirect costs, the direct costs will include the basic commodity costs, distribution costs, training costs, and human resource costs. G6PD testing will have direct and indirect effects on *P. vivax* malaria burden. By facilitating the use of primaquine the direct effects include (relapse) episodes of vivax averted and the costs associated with these. The indirect effects include the prevention of transmission, decreasing the incidence of new *P. vivax* infections. These are difficult to quantify due to our limited understanding of hypnozoites and the effect is likely to be highly heterogeneous.

#### Cost-effectiveness models

Economic modelling can be used to look at the long-term costs and outcomes of G6PD screening and primaquine use. For *P. vivax*, these models might include decision trees to look at the outcome of a single episode, which can be extended to Markov models to look at the impact of primaquine on recurrent episodes, or dynamic transmission models to investigate the impact of primaquine on transmission. In these models, costs and outcomes are linked to information on disease progression to produce incremental cost-effectiveness ratios, such as the cost per DALY averted or the cost per infection averted.

These models can be used to look at whether G6PD testing is cost-effective for a given population where either *P. vivax* infections are not being treated with primaquine or where primaquine is given without a G6PD test. In areas where primaquine is not currently being prescribed, a cost-effectiveness model will be important to demonstrate the long-term impact of primaquine on recurrences. Where primaquine is currently prescribed without G6PD testing, economic modelling could weigh the likelihood of haemolytic events against the burden of disease caused by recurrent vivax episodes. If haemolytic events are rare, it may be more economical to distribute primaquine without G6PD testing; however, medical staff must be comfortable implementing this policy choice. If health care providers have ethical concerns and are unwilling to take this risk, the economic justifications are not relevant.

Economic models could also be used to evaluate which of the aforementioned screening tests and strategies (Figs. 1, 2, 3) would be most cost-effective in a given setting. The biosensor and RDT tests have different diagnostic accuracies and costs. Instead of simply looking at which test is least expensive, an economic model will synthesize the evidence concerning the epidemiology of vivax and the prevalence and variant of G6PD deficiency to determine which strategy will provide the most cost-effective option in different settings. This evidence can enable the available malaria funding to be allocated more strategically.

#### Parameter values

Information needed for these models includes the prevalence of G6PDd in the population testing positive for vivax malaria, the probability of haemolysis or death when primaquine is given to a G6PD deficient individual, the probability of relapse if given or not given primaquine, adherence to primaquine treatment, and the costs of all screening and treatment components. While the cost of a G6PD test is often set low by manufacturers, the cost to the healthcare provider is often inflated due to procurement and logistics. This will need to be investigated by detailed costing research or through a sensitivity analysis in the model. Other costs that need better characterization are those for clinical care of vivax cases, and those for the management of severe haemolytic events [46]. At this point, limited information exists on many of the parameters needed to populate an economic model; moreover, a number of these parameters are likely to vary by setting. Further research is needed to reduce uncertainty and make these models informative.

In the future, economic modelling might capture wider implications of G6PD testing in other disease areas. G6PD deficiency is a common disorder and many other widely used drugs can cause haemolysis in G6PD deficient individuals. More data would be needed to populate such a model but this could lead to further economic justification for the implementation of G6PD testing.

### Health system integration: a review of four countries

A critical component to broader access and use of G6PD diagnostics is their integration into public health systems. Countries face a number of considerations when discussing implementation of a G6PD test including: *P. vivax* burden, elimination phase [47], income level, health budget, health system capacity to implement a new product and degree of training required for relevant staff. Using these factors, four countries (Bangladesh, Cambodia, Malaysia, and the Philippines; Tables 3, 4), across a variety of income, malaria burden and health capacity levels are reviewed here to assess the possibility and need to implement routine G6PD testing.

**Table 3:** Background Country Information

	Bangladesh	Cambodia	Malaysia	Philippines
PQ is used for radical treatment of *P.vivax* (in guidelines)	Yes (2008)	Yes (2015)	Yes	Yes (2007)
G6PD test is a requirement before treatment with PQ	No	Yes (2015)	Yes	Yes (2011)
Directly observed treatment with PQ is undertaken	No	No	Yes	Yes (2010)
# of confirmed malaria cases (all types) (year)	3864 (2013)	21,309 (2013)	3850 (2013)	6514 (2013)
% *P. vivax*	13 %	45 %	8 %	20 %
Treatment for vivax?	Cq + PQ 0.25 mg/kg (14 days)	DHA-PPQ + PQ 0.25 mg/kg (14 days)	CQ + PQ 0.5 mg/kg (14 days)	CQ + PQ 0.5 mg/kg (14 days)
Target Elimination Date	2030	2025	2020	2020

Source: WHO World Malaria Report 2014 and APMEN

**Table 4:** Background information malaria screening and treatment

Questions	Bangladesh	Cambodia	Malaysia	Philippines
What are the current screening procedures for Pv?	Microscopy and RDT at health facilities and community level	Microscopy and RDT at health facilities, RDT at community-level	Microscopy	Microscopy and RDTs. RDTs in rural health facilities by trained barangay health workers (BHWs). Microscopy in municipal health clinics and gov’t hospitals. Trained BHWs can also conduct microscopy
Active, passive, mass test or treat (MSAT), focused test and treat (FSAT)	Both active and passive case detection. Focused test and treat in pre-elimination areas	Passive case detection, some active detection with research. No MSAT or FSAT	Active and passive case detection	Passive case detection, some active detection
Is G6PD testing mandatory? Is it recommended prior to PQ administration?	Not mandatory	Mandatory testing and recommended prior to PQ	Mandatory testing prior to PQ. All newborns tested at birth	Testing is recommended
If G6PD testing is not mandatory, are their alternative procedures in place to assess and monitor risk of post-treatment haemolysis? Are patients monitored treatment adherence?	Patient is advised to report haemolysis. Follow ups are done by NGO-PR (BRAC supported consortium) at day 3, 7, and 14	No monitoring system in place	Patients are monitored	Patients are advised to report haemolysis. Treatment adherence is supposed to be monitored by BHW. No monitoring of haemolysis
Who performs the majority of screening activities?	Outreach Lab: Lab tech (NGO) Community Clinic: community health care provider (CHCP) Union sub-centers: medical officer, health assistant (GoB) Field Level: Health Assistant (GoB) or Shastho Kormi/Shastho Shebika (NGO) Medical officer at the District, Upazila and Tertiary facility level	Lab tech, nurse, or community health worker	Medical lab techs and nurses	MDs at clinics and hospitals Licensed midwives or BHW at rural health facilities

Malaysia has achieved a substantial reduction in malaria cases over the past several years, with 3850 cases reported in 2013, mostly concentrated in the Sabah and Sarawak regions of the country and the Central Highlands of Western Malaysia [47]. The country aims for elimination by 2017 and certification by 2020 [48]. Malaysia has had on-going malaria control and elimination strategies since the 1960s with original efforts at control beginning at the start of the 20th century. Currently, Malaysia uses internal budgeting to supply the resources necessary to combat malaria [48]. G6PD deficiency has been tackled through extensive use of the FST and G6PD deficiency tests are integrated into newborn screening programmes [49]. G6PD status is recorded and kept at the village and hospital level so that appropriate treatment can be provided to patients. A single FST costs an estimated $0.28 per test and $140 for the FST specific equipment (national malaria control programme, personal communication) within the country. Malaysia maintains FST kits at all hospitals and health clinics. All malaria cases diagnosed in the country are admitted to a hospital. Malaysia provides in-patient care to *P. vivax* malaria patients and 14 days of 0.25 mg/kg bw primaquine is provided to people with intermediate FST results (mild G6PD deficiency) compared to the 0.5 mg/kg bw to G6PD normal patients. Concerns over the need for observation of patients for adverse haemolytic events is limited and the exclusive use of a qualitative test format seems justified.

Routine G6PD testing in the Philippines started in 1998 in the context of a comprehensive new born screening programme and became mandatory in 2004. While the Philippine government now finances 39 % of the anti-malaria budget, with expectations to increase government budget coverage to 57 % from 2015 to 2017, the bulk of the remaining funding comes from the Global Fund [50] The country is seeking to integrate routine G6PD testing into their national health insurance programme and cover costs through internal resources (Fe Esperanza Espino, personal communication). Testing is conducted with FST at 5000 newborn screening centers across the country, with 20 confirmatory centers for all identified G6PD deficient individuals by spectrophotometry. The Philippine records indicate that approximately 2 % of the population is G6PD deficient [51]. The Philippines is restructuring funding of G6PD testing, with the imminent cessation of Global Fund support overlapping with national efforts to ramp up their domestic capabilities. Given the Philippines moderate domestic resources and expected strain on budgets during the transition out of Global Fund programmes in coming years, the country may be able to optimize the use of both RDTs and biosensors. Qualitative RDTs may be ideal for quick, PoC testing by primary care providers and village health workers, while biosensors could provide more detailed data for potentially heterozygous females or customized therapy. While the FST has successfully been implemented in the country, the superior operational characteristics of the Carestart RDT render the latter a potential candidate to replace the FST in the long term.

Cambodia and Bangladesh are not testing G6PD status on a routine basis [52]. In light of the haemolytic risk of primaquine Cambodia only recently recommended single dose primaquine treatment in *P. falciparum* cases without G6PD testing [29], while Bangladesh provides primaquine for radical cure on a regular basis. In the absence of alternative drugs to primaquine for hypnozoitocidal treatment Cambodia will eventually need to include the drug in its treatment schedules in order to pursue malaria elimination from the country [6]. Cambodia will only make primaquine radical cure for vivax available when it can test reliably for G6PDd.

Following current WHO guidelines [8] the practice of providing radical cure without routine G6PD testing calls for a comprehensive risk—benefit analysis within Bangladesh, surveys to assess the local prevalence of G6PDd are under way in parts of the country [51].

## Discussion and conclusion

The potential risks of 8-aminoquinoline therapy in the small subpopulation of G6PD deficient vivax patients have hindered the appropriate treatment of the large majority of vivax patients. The availability of affordable PoC tests for G6PDd is essential to detect at-risk patients and to optimize the management of vivax malaria. New G6PD tests are becoming available which may be able to help calibrate the most appropriate 8-aminoquinoline regimen for patients with vivax malaria. A quantitative, easy to use and handheld test device that can also provide a haemoglobin concentration would add great value. Current qualitative RDT formats can reliably classify hemizygous males and homozygous females, but may fail to correctly diagnose heterozygous females with intermediate G6PD activity. Nevertheless the format of a qualitative test is appealing, due to the similarity of the product to malaria RDTs, its ease of use and a performance comparable to the most routinely used test, the FST.

The roll out of G6PD testing will have implications in terms of costs. It will be important to understand these costs with different product concepts having different cost structures which will be defined by applied treatment algorithms, local *P. vivax* epidemiology and the local health system. Much of the data needed for these analyses are not yet available. Future research should include data collection appropriate for use in cost-effectiveness studies, which will be useful to inform different implementation modalities.

With increasing availability of PoC tests for G6PDd comes the potential to extend the coverage of radical cure and advance towards elimination of vivax malaria. To this end it is essential to strengthen the knowledge on how best to introduce and integrate G6PD tests into clinical practice based on cost and operational feasibility evidence.

## Additional files

**Additional file 1.** GeoDx. An online open source tool to guide G6PD diagnostics procurement.

## References

[1] Price RN, Tjitra E, Guerra CA, Yeung S, White NJ, Anstey NM (2007). Vivax malaria: neglected and not benign. Am J Trop Med Hyg.

[2] Mueller I, Galinski MR, Baird JK, Carlton JM, Kochar DK, Alonso PL (2009). Key gaps in the knowledge of *Plasmodium vivax*, a neglected human malaria parasite. Lancet Infect Dis..

[3] Carlton JM, Sina BJ, Adams JH (2011). Why is *Plasmodium vivax* a neglected tropical disease?. PLoS Neglect Trop Dis.

[4] Price RN (2014). Improving the radical cure of *Plasmodium vivax* malaria. Am J Trop Med Hyg.

[5] Nasveld PE, Edstein MD, Reid M, Brennan L, Harris IE, Kitchener SJ (2010). Randomized, double-blind study of the safety, tolerability, and efficacy of tafenoquine versus mefloquine for malaria prophylaxis in nonimmune subjects. Antimicrob Agents Chemother.

[6] Ashley EA, Recht J, White NJ (2014). Primaquine: the risks and the benefits. Malar J..

[7] von Seidlein L, Auburn S, Espino F, Shanks D, Cheng Q, McCarthy J (2013). Review of key knowledge gaps in glucose-6-phosphate dehydrogenase deficiency detection with regard to the safe clinical deployment of 8-aminoquinoline treatment regimens: a workshop report. Malar J..

[8] WHO (2015). Guidelines for the treatment of malaria.

[9] Domingo GJ, Satyagraha AW, Anvikar A, Baird K, Bancone G, Bansil P (2013). G6PD testing in support of treatment and elimination of malaria: recommendations for evaluation of G6PD tests. Malar J.

[10] MPAC (2015). Point-of-care G6PD testing to support safe use of primaquine for the treatment of vivax malaria.

[11] Manganelli G, Masullo U, Passarelli S, Filosa S (2013). Glucose-6-phosphate dehydrogenase deficiency: disadvantages and possible benefits. Cardiovasc Hematol Disord Drug Targets.

[12] Howes RE, Battle KE, Satyagraha AW, Baird JK, Hay SI (2013). G6PD deficiency: global distribution, genetic variants and primaquine therapy. Adv Parasitol.

[13] Beutler E (2008). Glucose-6-phosphate dehydrogenase deficiency: a historical perspective. Blood.

[14] Gomez-Manzo S, Terron-Hernandez J, la Mora-De De, la Mora I, Gonzalez-Valdez A, Marcial-Quino J, Garcia-Torres I (2014). The stability of G6PD is affected by mutations with different clinical phenotypes. Int J Mol Sci.

[15] Kotaka M, Gover S, Vandeputte-Rutten L, Au SW, Lam VM, Adams MJ (2005). Structural studies of glucose-6-phosphate and NADP+ binding to human glucose-6-phosphate dehydrogenase. Acta Crystallogr D Biol Crystallogr.

[16] WHO Working Group (1989). Glucose-6-phosphate dehydrogenase deficiency. Bull World Health Organ.

[17] Cappellini MD, Fiorelli G (2008). Glucose-6-phosphate dehydrogenase deficiency. Lancet.

[18] Minucci A, Moradkhani K, Hwang MJ, Zuppi C, Giardina B, Capoluongo E (2012). Glucose-6-phosphate dehydrogenase (G6PD) mutations database: review of the “old” and update of the new mutations. Blood Cells Mol Dis.

[19] Leslie T, Briceno M, Mayan I, Mohammed N, Klinkenberg E, Sibley CH (2010). The impact of phenotypic and genotypic G6PD deficiency on risk of plasmodium vivax infection: a case-control study amongst Afghan refugees in Pakistan. PLoS Med.

[20] Louicharoen C, Patin E, Paul R, Nuchprayoon I, Witoonpanich B, Peerapittayamongkol C (2009). Positively selected G6PD-Mahidol mutation reduces *Plasmodium vivax* density in Southeast Asians. Science.

[21] Luzzatto L, Seneca E (2014). G6PD deficiency: a classic example of pharmacogenetics with on-going clinical implications. Br J Haematol.

[22] Johnson MK, Clark TD, Njama-Meya D, Rosenthal PJ, Parikh S (2009). Impact of the method of G6PD deficiency assessment on genetic association studies of malaria susceptibility. PLoS One.

[23] Howes RE, Piel FB, Patil AP, Nyangiri OA, Gething PW, Dewi M (2012). G6PD deficiency prevalence and estimates of affected populations in malaria endemic countries: a geostatistical model-based map. PLoS Med.

[24] Mason PJ, Bautista JM, Gilsanz F (2007). G6PD deficiency: the genotype-phenotype association. Blood Rev.

[25] Meissner PE, Coulibaly B, Mandi G, Mansmann U, Witte S, Schiek W (2005). Diagnosis of red cell G6PD deficiency in rural Burkina Faso: comparison of a rapid fluorescent enzyme test on filter paper with polymerase chain reaction based genotyping. Br J Haematol.

[26] Chiu DT, Zuo L, Chao L, Chen E, Louie E, Lubin B (1993). Molecular characterization of glucose-6-phosphate dehydrogenase (G6PD) deficiency in patients of Chinese descent and identification of new base substitutions in the human G6PD gene. Blood.

[27] LaRue N, Kahn M, Murray M, Leader BT, Bansil P, McGray S (2014). Comparison of quantitative and qualitative tests for glucose-6-phosphate dehydrogenase deficiency. Am J Trop Med Hyg.

[28] Baird JK, Dewi M, Subekti D, Elyazar I, Satyagraha AW (2014). Noninferiority of glucose-6-phosphate dehydrogenase deficiency diagnosis by a point-of-care rapid test vs the laboratory fluorescent spot test demonstrated by copper inhibition in normal human red blood cells. Transl Res.

[29] Roca-Feltrer A, Khim N, Kim S, Chy S, Canier L, Kerleguer A (2014). Field trial evaluation of the performances of point-of-care tests for screening G6PD deficiency in Cambodia. PLoS One.

[30] Bancone G, Chu CS, Chowwiwat N, Somsakchaicharoen R, Wilaisrisak P, Charunwatthana P (2015). Suitability of capillary blood for quantitative assessment of G6PD activity and performances of G6PD point-of-care tests. Am J Trop Med Hyg.

[31] Nantakomol D, Paul R, Palasuwan A, Day NP, White NJ, Imwong M (2013). Evaluation of the phenotypic test and genetic analysis in the detection of glucose-6-phosphate dehydrogenase deficiency. Malar J.

[32] Ainoon O, Alawiyah A, Yu YH, Cheong SK, Hamidah NH, Boo NY (2003). Semiquantitative screening test for G6PD deficiency detects severe deficiency but misses a substantial proportion of partially-deficient females. Southeast Asian J Trop Med Public Health.

[33] Peters AL, Van Noorden CJ (2009). Glucose-6-phosphate dehydrogenase deficiency and malaria: cytochemical detection of heterozygous G6PD deficiency in women. J Histochem Cytochem.

[34] Shah SS, Diakite SA, Traore K, Diakite M, Kwiatkowski DP, Rockett KA (2012). A novel cytofluorometric assay for the detection and quantification of glucose-6-phosphate dehydrogenase deficiency. Sci Rep..

[35] PATH. A Guide to Fluorescnt Spot Testing for G6PD Deficiency. Programme for Appropriate Technology in Health, 2014.

[36] De Niz M, Eziefula AC, Othieno L, Mbabazi E, Nabukeera D, Ssemmondo E (2013). Tools for mass screening of G6PD deficiency: validation of the WST8/1-methoxy-PMS enzymatic assay in Uganda. Malar J..

[37] Tinley KE, Loughlin AM, Jepson A, Barnett ED (2010). Evaluation of a rapid qualitative enzyme chromatographic test for glucose-6-phosphate dehydrogenase deficiency. Am J Trop Med Hyg.

[38] Kim S, Nguon C, Guillard B, Duong S, Chy S, Sum S (2011). Performance of the CareStart G6PD deficiency screening test, a point-of-care diagnostic for primaquine therapy screening. PLoS One.

[39] Osorio L, Carter N, Arthur P, Bancone G, Gopalan S, Gupta SK (2015). Performance of BinaxNOW G6PD deficiency point-of-care diagnostic in *P. vivax*-infected subjects. Am J Trop Med Hyg.

[40] Baird JK, Surjadjaja C (2011). Consideration of ethics in primaquine therapy against malaria transmission. Trends Parasitol.

[41] Beutler E, Duparc S, G6PD Deficiency Working Group (2007). Glucose-6-phosphate dehydrogenase deficiency and antimalarial drug development. Am J Trop Med Hyg.

[42] Brewer GJ, Powell RD, Swanson SH, Alving AS (1964). Hemolytic effect of primaquine. Xvii. Hexokinase activity of glucose-6-phosphate dehydrogenase-deficient and normal erythrocytes. J Lab Clin Med.

[43] Kheng S, Muth S, Taylor WR, Tops N, Kosal K, Sothea K (2015). Tolerability and safety of weekly primaquine against relapse of *Plasmodium vivax* in Cambodians with glucose-6-phosphate dehydrogenase deficiency. BMC Med.

[44] Douglas NM, Nosten F, Ashley EA, Phaiphun L, van Vugt M, Singhasivanon P (2011). *Plasmodium vivax* recurrence following falciparum and mixed species malaria: risk factors and effect of antimalarial kinetics. Clin Infect Dis.

[45] Betuela I, Rosanas-Urgell A, Kiniboro B, Stanisic DI, Samol L, de Lazzari E (2012). Relapses contribute significantly to the risk of *Plasmodium vivax* infection and disease in Papua New Guinean children 1–5 years of age. J Infect Dis.

[46] Peixoto HM, Brito MA, Romero GA, Monteiro WM, de Lacerda MV, de Oliveira MR (2015). G6PD deficiency in male individuals infected by Plasmodium vivax malaria in the Brazilian Amazon: a cost study. Malar J..

[47] WHO (2014). World Malaria Report 2013.

[48] WHO (2015). Eliminating malaria: case study 8. Progress towards elimination in Malaysia.

[49] Leong YH, Gan CY, Tan MA, Majid MI (2014). Present status and future concerns of expanded newborn screening in malaysia: sustainability, challenges and perspectives. Malays J Med Sci.

[50] The Global Fund to Fight AIDS TaM. Philippines. 2015. http://portfolio.theglobalfund.org/en/Country/Index/PHL. Accessed 07.09.2015.

[51] APMEN. APMEN-PATH CONSULTATION: Strategies to implement G6PD screening to achieve successful radical cure of P. vivax in endemic countries. 2015. http://apmen.org/path-vivax-meeting-2015/. Accessed 07.09.2015.

[52] CNM. National treatment guidelines for malaria in Cambodia. Minister of Health, editor. Phnom Penh, 2015. http://whothailand.healthrepository.org/bitstream/123456789/1442/1/NTG%20in%20English-Final.pdf.

[53] von Fricken ME, Weppelmann TA, Eaton WT, Masse R, Madsen VE, De Rochars B (2014). Performance of the CareStart glucose-6-phosphate dehydrogenase (G6PD) rapid diagnostic test in Gressier, Haiti. Am J Trop Med Hyg..

