# Supplementary material for: The challenges of introducing routine G6PD testing into radical cure: a workshop report
Source: Malar J. 2015 Sep 29;14:377. doi: 10.1186/s12936-015-0896-8 (PMC4587750; doi:10.1186/s12936-015-0896-8)
Supplement: Supplementary file 1 — Additional file 1. GeoDx. An online open source tool to guide G6PD diagnostics procurement. [file 12936_2015_896_MOESM1_ESM.docx]

**Supplementary table - GeoDx**

| **GeoDx. An Online Open Source Tool to Guide G6PD Diagnostics Procurement**  To help guide the development and introduction of new diagnostics platforms throughout various regions, PATH is developing GeoDx, an interactive tool that provides a direct quantitative comparison of the financial costs associated with the deployment of different diagnostic platforms. The tool merges an interactive geospatial map, a detailed database of variables on epidemiology, health facility infrastructure, diagnostic product characteristics and cost structures. The user can set key parameters such as price per unit corresponding to a particular diagnostics test, allowing for quantitative comparison of the cost per facility of different diagnostic platforms. In its current configuration GeoDx can be used to compare two different G6PD diagnostic product concepts: one consisting of a device and disposable, similar to the biosensor, and the other being only a disposable, such as a RDT. The outputs correlate total commodity costs to facility type and disease incidence. |
| --- |
